# Genome-Wide Association Studies Reveal Neurological Genes for Dog Herding, Predation, Temperament, and Trainability Traits

**DOI:** 10.3389/fvets.2021.693290

**Published:** 2021-07-21

**Authors:** Shuwen Shan, Fangzheng Xu, Bertram Brenig

**Affiliations:** Department of Animal Sciences, Faculty of Agricultural Sciences, Institute of Veterinary Medicine, University of Goettingen, Göttingen, Germany

**Keywords:** dog behavior, GWAS, herding, predation, temperament, trainability, neurological genes

## Abstract

Genome-wide association study (GWAS) using dog breed standard values as phenotypic measurements is an efficient way to identify genes associated with morphological and behavioral traits. As a result of strong human purposeful selections, several specialized behavioral traits such as herding and hunting have been formed in different modern dog breeds. However, genetic analyses on this topic are rather limited due to the accurate phenotyping difficulty for these complex behavioral traits. Here, 268 dog whole-genome sequences from 130 modern breeds were used to investigate candidate genes underlying dog herding, predation, temperament, and trainability by GWAS. Behavioral phenotypes were obtained from the American Kennel Club based on dog breed standard descriptions or groups (conventional categorization of dog historical roles). The GWAS results of herding behavior (without body size as a covariate) revealed 44 significantly associated sites within five chromosomes. Significantly associated sites on CFA7, 9, 10, and 20 were located either in or near neuropathological or neuronal genes including *THOC1, ASIC2, MSRB3, LLPH, RFX8*, and *CHL1*. *MSRB3* and *CHL1* genes were reported to be associated with dog fear. Since herding is a restricted hunting behavior by removing killing instinct, 36 hounds and 55 herding dogs were used to analyze predation behavior. Three neuronal-related genes (*JAK2, MEIS1*, and *LRRTM4*) were revealed as candidates for predation behavior. The significantly associated variant of temperament GWAS was located within *ACSS3* gene. The highest associated variant in trainability GWAS is located on CFA22, with no variants detected above the Bonferroni threshold. Since dog behaviors are correlated with body size, we next incorporate body mass as covariates into GWAS; and significant signals around *THOC1, MSRB3, LLPH, RFX8, CHL1, LRRTM4*, and *ACSS3* genes were still detected for dog herding, predation, and temperament behaviors. In humans, these candidate genes are either involved in nervous system development or associated with mental disorders. In conclusion, our results imply that these neuronal or psychiatric genes might be involved in biological processes underlying dog herding, predation, and temperament behavioral traits.

## Introduction

Dogs are man's best friend and the first domesticated animal, originating from a now-extinct wolf population. Dogs have shared living space and food sources with humans and have maintained this close relationship for more than 11,000 years (1). For only 200–300 years, humans have selectively bred dogs for excellence in herding, hunting, and obedience and have created diverse breeds with a wealth of behaviors. At the same time, humans have also bred dogs for different morphological traits such as body types, sizes, skull shapes, coat colors, and textures according to human preferences and needs. Two major bottlenecks in dog history, i.e., early domestication and the creation of modern breeds, have characterized long-range linkage disequilibrium (LD) within dog breeds, providing an excellent natural model for studying morphology, complex diseases, and behaviors (2). Over the past two decades, scientists have attempted to explain the genetic basis of phenotypic variation among dog breeds. Many cross-breed researches were performed including morphologic traits (3–5), diseases (6), behavior or cognition (6–8), and athletic ability (9).

Dog behavior traits have been reported to be highly heritable, with a mean among-breed heritability (h^2^) of 0.51 ± 0.12 (standard deviation) for 14 behavioral traits. Specifically, high h^2^ values were observed for attachment and attention-seeking (0.56), chasing (0.62), stranger-directed aggression (0.68), and trainability (0.73) (7). However, the genetic mapping of behavior among dog breeds remains challenging. One reason is that behavior and cognition are complex traits, which are difficult to define and measure accurately (10). Therefore, different methods have been developed to classify and describe behavioral phenotypes. Behavioral studies across and within dog breeds have been explored and discussed. With the use of large single-nucleotide polymorphism (SNP) datasets and C-BARQ data of diverse breeds, dog fearlessness and aggression traits have been mapped to be associated with *GNAT3*-*CD36* (CFA18) and *IGSF1* (CFAX) loci (8). In the same study, variants within body size genes (*IGF1* and *HMGA2*) showed significant associations with dog behaviors such as dog rivalry, separation anxiety, touch sensitivity, and owner-directed aggression (8). In one recent study, using breed-averaged C-BARQ data as phenotypes, 131 SNPs were demonstrated to be significantly associated with dog behavioral differences among 101 breeds, and the identified neurological candidate genes were highly expressed in the brain (7). In addition, the among-breed heritability of 14 behavioral traits was significantly higher than the heritability assessed in large within-breed samples (7). This study only did genome-wide association study (GWAS) considering body size covariates, and loci that affect both body size and behavior might have been missed. Another GWAS of dog cognition (with and without body mass factors) using breed-averaged phenotypic values identified five SNPs significantly associated with breed differences in dog communication, memory, inhibitory control, and physical reasoning and identified 188 genes related to breed cognitive differences (6).

Behavioral traits often exhibit complexity, polygenic control, and susceptibility to environmental influences. And they are inherited in linkage with other traits; for example, behavioral traits in dog are related to body size (11). For some behavioral studies, within-breed studies have shown good results and have been able to obtain more specific behavioral or cognitive loci. Recently, using the C-BARQ data as phenotypes, 11 SNPs within eight genomic regions were detected to be significantly related with six canine personality traits in Labrador retrievers (12). Two chromosome regions of CFA7:75–79 Mb and CFA20:8–11 Mb were investigated to be significantly associated with fearfulness in German Shepherd (13). Meanwhile, a locus of CFA11:12.8 Mb was found to be significantly associated with fearfulness when investigated in Great Dane (14). These regions and the contained genes all correspond to the neuropsychiatric or neuronal gene regions in humans. In addition, human obsessive-compulsive disorder (OCD) has phenotypes similar to those in canine compulsive disorder (CCD), such as repetitive and time-consuming behaviors (15). Four CCD candidate genes, i.e., *CDH2, CTNNA2, ATXN1*, and *PGCP*, were mapped by case–control GWAS in Doberman pinschers and validated in high-risk breeds (16). Structural variants on CFA6 containing *GTF2I* and *GTF2IRD1* genes could contribute to behavioral differences (extreme sociability) between dogs and wolves, and these two genes are associated with human Williams–Beuren syndrome, which is characterized by a happy and friendly disposition (17). Notably, *HS6ST2* gene was first reported to be associated with dog sociability behavior (8) and recently was detected to be significantly related to human neuroticism in GWAS of 405,274 UK Biobank samples (18). This indicates that dogs could be good natural models for studying the molecular etiology of human neural disorders.

Herding dogs were bred to help people manage livestock, and they excel at controlling livestock movement. Herding derives from predatory behavior by amplifying some predatory instincts such as eye staring, stalking, and chasing while suppressing other instincts as crush, bite, or kill the prey (19). Herding dogs are energetic, enthusiastic, and eager to work. If they are not properly trained or assigned tasks, they even use the inclination to herd other creatures including human beings (20). They also exhibit characteristics such as agility, bravery, steadiness, and relatively low aggressiveness (21). The current study used breed-specific behaviors and groupings from the American Kennel Club (AKC), the most authoritative organization for the registration and classification of purebred dogs in the United States. The AKC recognizes and classifies 197 modern purebred dog breeds into seven loosely defined groups based on their breed features (heritage, physical attributes, and behavior) and historical roles: sporting, hound, working, terrier, toy, non-sporting, and herding groups (22). The AKC group method has been successfully applied in identifications of genetic factors contributing to athleticism in sporting and hound dogs (9) as well as relationship investigations between artificial selection and human-directed play behavior (23). Genetic mapping of dog herding behavior has been first studied as qualitative variable in 148 dog breeds (24); and three other dog behaviors including pointing, boldness, and trainability were studied using cross-breed mapping.

Different dog breed specific traits are selected based on different human purposes; thus, each dog breed has its unique temperament and trainability characteristic. Temperament is of great importance for dog breeding, especially in choosing good guide dogs (25). Pet owners are also interested in matching dog with suitable temperament (26). Among the genetic studies of temperament traits, dog activity-impulsivity endophenotype was first studied through the association analysis of candidate gene *DRD4* (27). Trainability levels were detected to have significant differences between seven breed groups (conventional breed categories), which implies that dog behavior traits such as trainability and boldness are partly caused by original function of breed. In the same study, scores of trainability, boldness, calmness, and dog sociability were all detected with significant differences among dog breeds (28). These breed-level behavioral differences can be used as phenotypes to study underlying genetic mechanisms, which will help us understand how these behaviors developed in dogs.

Significant brain neuroanatomical variations among breeds with different behavioral specialties, such as herding, hunting, guarding, and companionship, are likely due to human selection for the behavior (29). It is reasonable to hypothesize that using a cross-breed research strategy could help us find loci that control significant behavioral variations between breeds. Therefore, this study used behavioral groupings provided by the AKC to perform cross-breed GWAS to find genetic markers associated with behavioral differences among breeds. Incorporating body size factors into dog behavior GWAS can bring both merits and drawbacks, as body size-related variants could also play roles in behaviors through their effects on brain architectures (30), while controlling body size factors could reveal genetic variants that are not explained by the brain or body size (7). Inspired by Gnanadesikan et al. (6), significant signals identified in GWASs either with or without body mass corrections were regarded as candidates in our analysis. This study provides clues to the molecular genetic mechanisms underlying canine behaviors such as herding, predation, temperament, and trainability. Understanding the formation of breed-specific behaviors in dogs will also pave the way for further elucidations of mechanisms underlying human neuropsychiatric disorders.

## Materials and Methods

### Samples and Phenotypes

**Figure 1:**
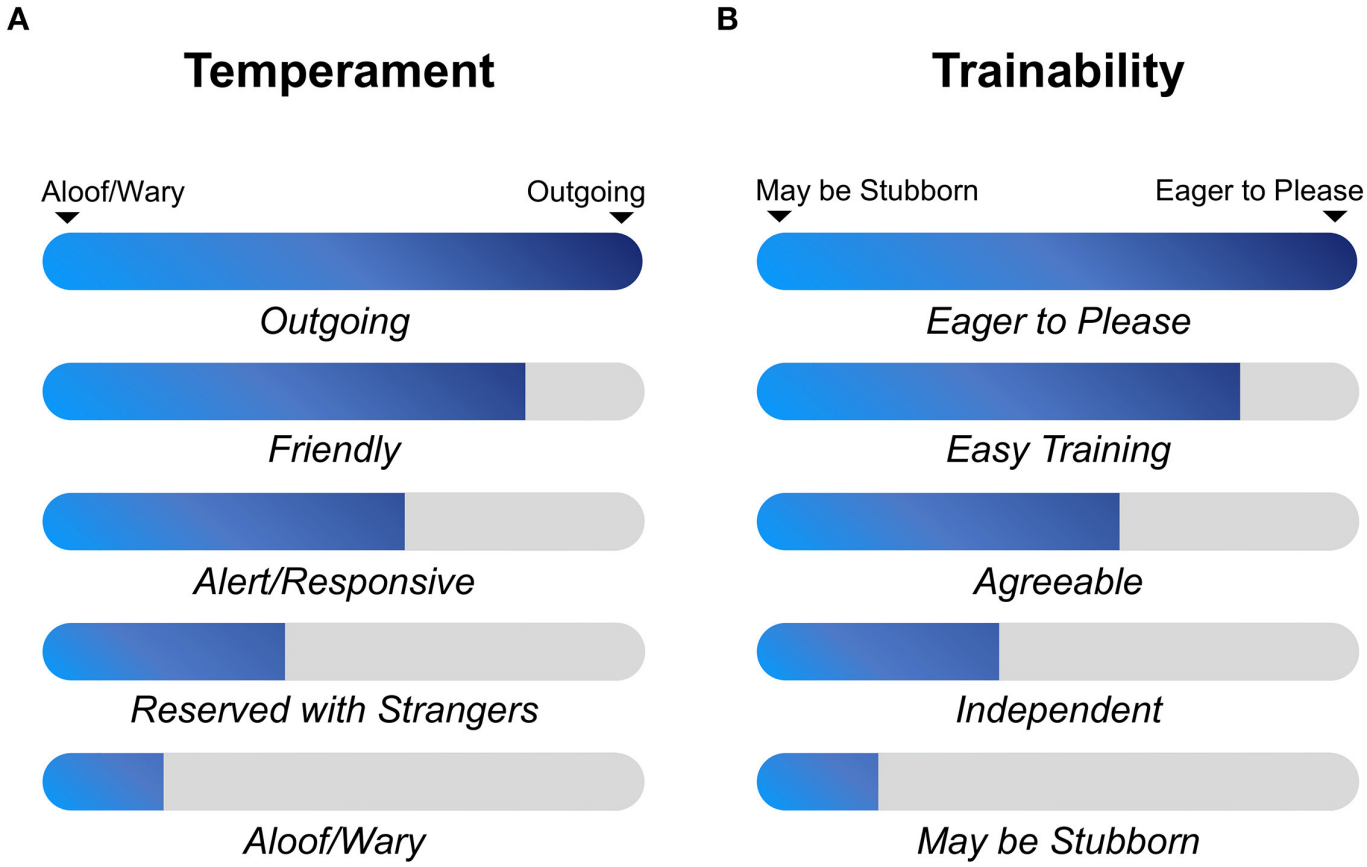
Classifications of trainability and temperament traits among modern dog breeds. Trait level information is obtained from AKC website (https://www.akc.org/dog-breeds/) (accessed on 20 December 2020), and each breed has a specific score for one of these five phenotype levels. **(A)** Aloof/wary, reserved with strangers, alert/responsive, friendly and outgoing were used to describe temperament character of each dog breed stereotype. **(B)** May be stubborn, independent, agreeable, easy training and eager to please were applied to describe trainability character of each dog breed stereotype.

All 268 whole-genome sequences of dogs that were used in this study have been extracted from vcf file data of 722 canine individuals (https://www.ncbi.nlm.nih.gov/bioproject/PRJNA448733), which is deposited by Dr. Elaine A. Ostrander group of the National Institutes of Health (NIH) (5). Many sources (*n* = 128) such as NIH Intramural Sequencing Center are involved in the data generation with funds such as Intramural Program of the National Human Genome Research Institute. The 268 dog genomes consist of 130 established dog breeds (Supplementary Table 1), and the selection criterion is same as described in (5). For herding behavior, dogs were divided into cases and controls according to whether they belong to the AKC herding group (conventional categorization) (https://www.akc.org/dog-breeds/herding/) or not. Forty-three herding group dogs were obtained, containing 15 modern dog breeds (Supplementary Table 1). In addition, an extra six modern dog breeds with a herding phenotype (Rottweiler, Bernese Mountain Dog, Fonni's Dog, Lapponian Herder, Samoyed, and Swedish Lapphund) were selected according to the article (24), although these breeds were classified to working group in the AKC. Twelve cases were obtained in this step, and finally 55 herding dogs were available as a case group. Herding dogs and hunting dogs are selected to meet different job requirements, and therefore, they have different degrees of prey-driven instincts. Thus, hounds and herding dogs can serve as good cases and controls for studying hunting behavior, such as aggressive behavior. In order to decipher this complex behavior, 36 hound group dogs were set as cases and 55 herding group dogs were regarded as controls for GWAS. Temperament and trainability traits were referred to the average scores of the AKC breed standard. When the ideal physical characteristics and temperament of a dog breed are specified in a written document, the breed becomes the standard breed. Therefore, different dog breeds have different levels of temperament (outgoing, friendly, alert/responsive, reserved with strangers, and aloof/wary) (Figure 1A) and trainability (eager to please, easy training, agreeable, independent, and may be stubborn) (Figure 1B). Since kennel club group classifications are not the most accurate way to apply those phenotypes, we only set the top two levels as cases and last two levels as controls, and the middle levels (agreeable and alert/responsive) were not included in GWAS analysis and considered as missing (NA). In total, 105 cases and 81 controls for the temperament analysis and 98 cases and 85 controls for trainability analysis were finally obtained (Table 1). Phenotype information of dog breed temperament and trainability traits were collected on 20 December 2020.

**Table 1:** Summary of dog behavioral phenotypes used in genome-wide association study (GWAS) analysis.

**Trait**	**Phenotype levels**	**Numbers of dog**	**Group**
Herding	Herding behavior	55	Case
	Non-herding	213	Control
Predation	Hound group	36	Case
	Herding group	55	Control
Temperament	Outgoing	19	Case
	Friendly	86	Case
	Alert/responsive	65	NA
	Reserved with strangers	76	Control
	Aloof/wary	5	Control
Trainability	Eager to please	63	Case
	Easy training	35	Case
	Agreeable	72	NA
	Independent	68	Control
	May be stubborn	17	Control

### Genome-Wide Association Study Analysis

To obtain high-quality and only biallelic variants [single-nucleotide variants (SNVs) and small indels] for GWAS, vcf file of 722 dog genomes was first filtered by PLINK 1.90 with the following functions: –max-alleles 2, –min-alleles 2, –minQ 20, –max-missing 0.9 (31). Then individual dogs for each GWAS were extracted from the above-filtered vcf file, and variants with missing value >1% (–maf 0.01) were removed using PLINK 1.90 (31). After filtering, 14,489,548, 14,654,804, 14,984,476, and 14,853,066 biallelic variants were used for GWASs of herding, predation, temperament, and trainability traits, respectively.

GWAS was conducted by applying a univariate linear mixed model with sex and kinship (relatedness matrix) as covariates. The model is available in GEMMA 0.98, and two steps of calculation were applied (32). A centered relatedness matrix was calculated in the first step, which was used as a covariate to adjust for sample structure after eigen-decomposition in the second step (32). The Wald test was applied for the association significance assessment. Bonferroni thresholds [*P*_*bon*_ = –log(0.05/number of analyzed variants)] were used to identify significant association sites for herding (*P*_*bon*_ = 8.46) and predation (*P*_*bon*_ = 8.47) behavior. As no associated variants were above Bonferroni thresholds for temperament and trainability, suggestive thresholds [*P*_*sug*_ = –log(1/number of analyzed variants)] of temperament (*P*_*sug*_ = 7.18) and trainability (*P*_*sug*_ = 7.17) were applied. The suggestive threshold was first introduced by Lander and Kruglyak (33), which represents one false positive that is expected per genome scan under the null hypothesis. Manhattan and quantile–quantile (QQ) plots were generated by qqman package (34). To account for body size factors in dog behavior, dog standard breed weight (SBW) and height (SBH) were further included in GWASs as covariates. The average values of body size were collected from (5). Only dog breeds that have SBW and SBH values were chosen for further analysis. The variant filtering conditions are the same as above. After filtering, 255 individuals with 14,416,697 variants, 88 dogs with 14,542,561 variants, 178 dogs with 14,829,902 variants, and 177 dogs with 14,726,409 variants were analyzed in herding (*P*_*bon*_ = 8.46, *P*_*sug*_ = 7.16), predation (*P*_*bon*_ = 8.46, *P*_*sug*_ = 7.16), temperament (*P*_*bon*_ = 8.47, *P*_*sug*_ = 7.17), and trainability (*P*_*bon*_ = 8.47, *P*_*sug*_ = 7.17) GWASs, respectively. Bonferroni and suggestive thresholds were shown in the figures of GWAS results.

The genomic inflation factor lambda (λ) was calculated with the following formula: λ = median (qchisq(1 – p, 1))/qchisq(0.5, 1) where p is a vector of *p*-values in GWAS results. The lambda inflation factor indicates the rate of excess false positive and the extent of the bulk inflation. When values of λ <1.1 are obtained, significant population stratification will not be considered, which was also observed in the GWAS of canine complex traits (35). The QQ plot shows the observed vs. expected –log *p*-values. The straight line in the QQ plot indicates the distribution of variant markers under the null hypothesis, and the skew at the right edge indicates those markers that are more strongly associated with the trait than would be expected by chance.

The detected associated signals were annotated by National Center for Biotechnology Information (NCBI) *Canis lupus* familiaris 3.1 Annotation Release 105. The positions were viewed by Genome Data Viewer with CanFam3.1 reference genome (https://www.ncbi.nlm.nih.gov/genome/gdv/?org=canis-lupus-familiaris).

### Alternative Allele Frequencies of Significantly Associated Variants

The allele frequencies of significantly associated sites were investigated in cases and controls for each GWAS trait setting separately using VCFtools 0.1.16 (36). The results of altered allele frequencies within these traits are shown in Table 2.

**Table 2:** Genome-wide association study (GWAS) significant associated variants of dog herding, predation, temperament, and trainability behavior traits.

**Trait**	**Variant**	**Ref**	**Alt**	**Alt_Freq_cases**	**Alt_Freq_controls**	***p*_value_a**	***p*_value_b**	**Nearest gene symbol**	**Distance to gene (bp)**
Herding	**CFA6:40747205**	**A**	**C**	**0.1545**	**0**	**2.20E−09**	**2.75E−09**	***LOC611691***	**0**
	**CFA6:41114381**	**T**	**C**	**0.1545**	**0**	**2.20E−09**	**2.75E−09**	***OR28H03***	**13,451**
	**CFA7:67137186**	**T**	**G**	**0.1759**	**0.0024**	**5.72E−10**	**2.07E−09**	***THOC1***	**594**
	**CFA7:67155662**	**C**	**G**	**0.1455**	**0.0023**	**6.50E−10**	**2.07E−09**	***THOC1***	**19,070**
	CFA7:67163810	A	T	0.1132	0.0023	2.78E−09	1.03E−08	*THOC1*	27,218
	CFA9:40067785	A	G	0.0545	0	2.33E−09	4.03E−09	*ASIC2*	0
	CFA9:40068138	T	C	0.0545	0	2.33E−09	4.03E−09	*ASIC2*	0
	**CFA10:8016660**	**A**	**G**	**0.8182**	**0.331**	**3.29E−09**	**1.33E−09**	***MSRB3***	**0**
	**CFA10:8116174**	**T**	**G**	**0.7455**	**0.2379**	**2.04E−09**	**3.66E−10**	***LOC111097584***	**32,715**
	**CFA10:8116175**	**T**	**C**	**0.7455**	**0.2402**	**1.73E−09**	**3.00E−10**	***LOC111097584***	**32,716**
	**CFA10:8116176**	**C**	**G**	**0.7455**	**0.239**	**1.38E−09**	**3.00E−10**	***LOC111097584***	**32,717**
	**CFA10:8581163**	**C**	**T**	**0.5091**	**0.1056**	**4.40E−10**	**9.63E−10**	***LLPH***	**50,728**
	**CFA10:8583785**	**T**	**A**	**0.5**	**0.1056**	**1.26E−09**	**2.54E−09**	***LLPH***	**48,106**
	CFA10:8589159	A	G	0.4909	0.1033	2.05E−09	3.87E−09	*LLPH*	42,732
	**CFA10:8597348**	**G**	**A**	**0.4909**	**0.1033**	**1.52E−09**	**2.80E−09**	***LLPH***	**34,543**
	**CFA10:8601766**	**C**	**T**	**0.5**	**0.1056**	**5.50E−10**	**1.10E−09**	***LLPH***	**30,125**
	**CFA10:8604778**	**C**	**T**	**0.5091**	**0.1132**	**3.67E−10**	**1.49E−10**	***LLPH***	**27,113**
	**CFA10:8614536**	**CG**	**C**	**0.5545**	**0.1479**	**2.00E−09**	**4.86E−10**	***LLPH***	**17,355**
	**CFA10:8614872**	**AAGCTC**	**A**	**0.5545**	**0.1479**	**2.00E−09**	**4.86E−10**	***LLPH***	**17,091**
	**CFA10:8615480**	**G**	**A**	**0.5545**	**0.1479**	**2.00E−09**	**4.86E−10**	***LLPH***	**16,411**
	**CFA10:41504918**	**C**	**CCCTTT**	**0.1636**	**0.0236**	**2.98E−10**	**1.03E−09**	***RFX8***	**0**
	**CFA10:41505049**	**T**	**A**	**0.1545**	**0.0235**	**1.56E−10**	**5.89E−10**	***RFX8***	**0**
	CFA10:41506217	A	G	0.1636	0.0258	2.70E−09	8.97E−09	*RFX8*	0
	CFA10:41506301	C	T	0.1636	0.0258	2.70E−09	8.97E−09	*RFX8*	0
	CFA10:41506568	C	T	0.1545	0.0235	2.31E−09	7.11E−09	*RFX8*	0
	CFA10:41506655	T	C	0.1636	0.0258	2.70E−09	8.97E−09	*RFX8*	0
	CFA10:41506849	C	T	0.1545	0.0235	2.31E−09	7.11E−09	*RFX8*	0
	CFA10:41507558	G	A	0.1636	0.0259	2.71E−09	8.99E−09	*RFX8*	0
	**CFA20:16594598**	**C**	**T**	**0.1364**	**0**	**8.48E−11**	**3.16E−10**	***LOC111091431***	**9,375**
	**CFA20:16595519**	**T**	**C**	**0.1364**	**0**	**8.48E−11**	**3.16E−10**	***LOC111091431***	**8,454**
	**CFA20:16595717**	**G**	**A**	**0.1364**	**0**	**8.48E−11**	**3.16E−10**	***LOC111091431***	**8,256**
	**CFA20:16595938**	**C**	**T**	**0.1364**	**0**	**8.48E−11**	**3.16E−10**	***LOC111091431***	**8,035**
	**CFA20:16596248**	**A**	**AAAG**	**0.1364**	**0**	**8.48E−11**	**3.16E−10**	***LOC111091431***	**7,725**
	CFA20:16596343	A	G	0.1296	0	2.48E−09	6.74E−09	*LOC111091431*	7,630
	CFA20:16596466	C	T	0.1273	0	1.65E−09	5.14E−09	*LOC111091431*	7,507
	**CFA20:16596631**	**A**	**G**	**0.1364**	**0**	**8.48E−11**	**3.16E−10**	***LOC111091431***	**7,342**
	**CFA20:16597311**	**C**	**T**	**0.1455**	**0**	**1.30E−10**	**4.84E−10**	***LOC111091431***	**6,662**
	**CFA20:16598698**	**C**	**G**	**0.1364**	**0**	**8.48E−11**	**3.16E−10**	***LOC111091431***	**5,275**
	**CFA20:16603809**	**A**	**C**	**0.1364**	**0**	**8.48E−11**	**3.16E−10**	***LOC111091431***	**164**
	**CFA20:16604304**	**T**	**G**	**0.1364**	**0**	**8.48E−11**	**3.16E−10**	***LOC111091431***	**0**
	**CFA20:16607008**	**A**	**T**	**0.1364**	**0**	**8.48E−11**	**3.16E−10**	***LOC111091431***	**0**
	**CFA20:16607290**	**T**	**C**	**0.1364**	**0**	**8.48E−11**	**3.16E−10**	***LOC111091431***	**0**
	**CFA20:16610276**	**C**	**T**	**0.1364**	**0**	**8.74E−11**	**3.28E−10**	***LOC111091431***	**0**
	**CFA20:16610335**	**G**	**A**	**0.1364**	**0**	**8.48E−11**	**3.16E−10**	***LOC111091431***	**0**
Predation	CFA1:93319503	C	T	0.1528	0.8273	2.59E−09	1.64E−06	*JAK2*	1,552
	CFA1:93319523	C	CATG	0.1528	0.8273	2.59E−09	1.64E−06	*JAK2*	1,532
	CFA1:93319862	T	C	0.1667	0.8364	1.28E−09	1.02E−06	*JAK2*	1,193
	CFA10:65924498	T	C	0.2639	0.9091	3.96E−10	6.02E−09	*MEIS1*	25,784
	CFA10:65924663	G	A	0.2639	0.9091	3.96E−10	6.02E−09	*MEIS1*	25,949
	CFA10:65924694	C	G	0.2639	0.9091	3.96E−10	6.02E−09	*MEIS1*	25,980
	CFA10:65924801	G	A	0.2639	0.9091	3.96E−10	6.02E−09	*MEIS1*	26,087
	CFA10:65925175	C	G	0.2639	0.9091	3.96E−10	6.02E−09	*MEIS1*	26,461
	**CFA17:47109846**	**C**	**T**	**0.3056**	**0.8182**	**2.97E−09**	**4.16E−10**	***LRRTM4***	**312,739**
	**CFA17:47109848**	**C**	**T**	**0.3056**	**0.8182**	**2.97E−09**	**4.16E−10**	***LRRTM4***	**312,741**
	**CFA17:47109850**	**T**	**A**	**0.3056**	**0.8182**	**2.97E−09**	**4.16E−10**	***LRRTM4***	**312,743**
	**CFA17:47109882**	**C**	**T**	**0.3056**	**0.8182**	**2.97E−09**	**4.16E−10**	***LRRTM4***	**312,775**
Temperament	**CFA15:23340008**	**A**	**T**	**0.7019**	**0.284**	**1.54E−08**	**1.92E−09**	***ACSS3***	**0**
Trainability	CFA22:34873149	A	G	0.5941	0.1633	5.94E−08	7.92E−08	*LOC111091672*	19,895

*Bold indicates significantly associated variants that were identified in GWASs both without and with body size as a covariate*.

*p_value_a, GWASs without body size as a covariate; p_value_b, GWASs with body size as a covariate*.

### Linkage Disequilibrium Analysis of Genome-Wide Association Study Significant Association Signals

LD of each significantly associated site was analyzed by PLINK 1.90 (31) using the following functions: –ld-window-kb 5,000, –ld-window 99,999, –ld-window-*r*^2^ 0.8. Sites with *r*^2^-value more than 0.8 are listed in Supplementary Table 2. Genes near or located around these LD sites were annotated by Genome Data Viewer.

### Analysis of Private Variants in Dogs With Herding Behavior

We next analyzed variants that were only present in 55 dogs with herding behavior. First, a total of 268 samples were quality controlled for all types of variants using VCFtools 0.1.16 (36). Only variants with minor allele frequency (MAF) >0.05, genotype quality (GQ) score > 20, and mean depth values >10x were selected. After separate filtering, 10,415,191 variants of 213 control dogs and 9,864,535 variants of 55 herding behavior dogs remained for further analysis. Private variants were analyzed by comparing the above-filtered vcf files of 55 herding dogs and 213 controls using “–diff-site” function in VCFtools 0.1.16 (36). The private variants were further annotated by SnpEff 5.0 with Ensembl genome 101 release (37). We have acquired 987,046 sites that were absent or rare (MAF <0.05) in non-herding controls, and these variants were present in at least one herding dog. Variants within protein-coding genes were selected for further analysis. Variants with possible functions (high and moderate impact) in protein-coding genes were chosen, and 611 high impact variants within 270 genes and 6,740 moderate-impact variants within 2,133 genes were left. After merging genes of high and moderate impacts, 2,287 private genes remained. Gene Ontology (GO) analysis was performed using these 2,287 genes with the online software WebGestalt [http://www.webgestalt.org/; (38)]. The top 10 significant biological processes and cellular components were chosen for further analysis; WebGestalt applied the false discovery rate (FDR) method to account for multiple testing.

The variant filtered quality conditions such as MAF and mean depth values could influence the variant content of filtered vcf files of cases and controls. For example, one variant has a MAF of 0.049 in 213 controls, while its MAF is 0.051 in 55 cases; then it will be one private variant because it is absent in quality-filtered vcf file of 213 controls due to MAF <0.05. To prioritize the private candidate variants, in these possibly functional private variants, their altered allele frequencies were further checked in raw vcf files of 55 herding and 213 control dogs separately using “–freq” function in VCFtools 0.1.16 (36). Variants present in more than one herding dog but not in controls, or variants with altered allele frequency differences >0.1 between cases and controls, are listed (Supplementary Table 3).

### Investigate Gene Expressions of 10 Candidate Genes in Online Gene Expression Databases

Gene expressions of 10 candidate genes (*THOC1, ASIC2, MSRB3, LLPH, RFX8, CHL1, JAK2, MEIS1, LRRTM4*, and *ACSS3*) were further examined by online database SCDevDB (https://scdevdb.deepomics.org) for single-cell atlas in the human neural developmental pathway (39). The cell types were oocyte, zygote, 2-cell, 4-cell, 8-cell, 16-cell, blastocyst, human embryonic stem cells (hESC), H1_24_wells, H1_96_wells, neural_D12 (neural cells generate from H1 cell line, 12 days after differentiation), neural_D26 (neural cells generate from H1 cell line, 26 days after differentiation), neural_D54 (neural cells generate from H1 cell line, 54 days after differentiation), and neural_D80 (neural cells generate from H1 cell line, 80 days after differentiation). Cell details are available at https://scdevdb.deepomics.org/data-summary/; data information of neural cell lines was referenced in (40).

These genes were further investigated in Allen Developing Mouse Brain Atlas [http://developingmouse.brain-map.org; (41)]. Days of embryonic (E) specimen age and postnatal (P) specimen age, which is relative to birth (P0), are used to define the mouse brain development stages.

## Results

### Distribution and Allele Frequencies of Genome Wide Association Study Associated Sites of Dog Herding, Predation, Temperament, and Trainability Traits

#### Genome-Wide Association Studies Not Including Body Size as a Covariate

**Figure 2:**
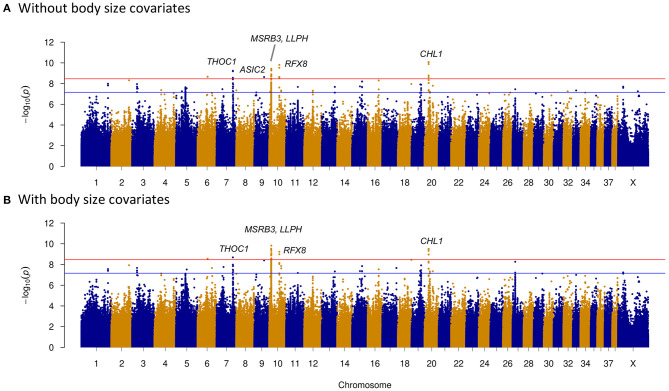
Manhattan plots of herding behavior genome-wide association study (GWAS). The plots show the –log10 *p*-values for all variants of GWAS. Red horizontal line represents the Bonferroni genome-wide significance threshold, and blue horizontal line indicates suggestive significance threshold. **(A)** Without including body size as a covariate. Candidate genes around significantly associated sites (above the Bonferroni threshold) were marked with red color in the Manhattan plot; they are *THOC1* of CFA7; *ASIC2* of CFA9; *MSRB3, LLPH*, and *RFX8* of CFA10; and *CHL1* of CFA20. **(B)** Including body size as a covariate. Candidate genes (*THOC1, MSRB3, LLPH, RFX8*, and *CHL1*) were marked in the plot.

We investigated four dog behavioral trait phenotypes (herding, predation, temperament, and trainability) using a univariate linear mixed model incorporating in GEMMA 0.98 (32). Sex and relatedness matrices (correcting for population stratification) were used as covariates to perform association tests on one single trait phenotype. For GWAS of dog herding behavior, 55 dogs with herding behavior and 213 control dogs were used. Forty-four significantly associated variants within regions of five chromosomes (CFA6, CFA7, CFA9, CFA10, and CFA20) were above the Bonferroni threshold (Figure 2). The most significantly associated region is located on CFA20 (16594598–16610335) including 16 associated sites, all of which were near or in one long non-coding RNA (lncRNA): *LOC111091431*. Five of them were within *LOC111091431*, and one variant at position 16,607,008 was located in the exonic region of the lncRNA. Moreover, this variant (CFA20:16607008 A>T) was only present in dogs with herding behavior (Table 2). Another variant (CFA20:16603809 A>C) was located only 164 bp upstream of *LOC111091431*. More importantly, *LOC111091431* is located 159,124 bp upstream of the neural cell adhesion molecule L1-like protein (*CHL1*), a neural-associated gene. On CFA7, one variant was 594 bp upstream of THO Complex 1 (*THOC1*) gene. One, two, and eight significantly associated intron variants were detected in acid sensing ion channel subunit 2 (*ASIC2*), methionine sulfoxide reductase B3 (*MSRB3*), and regulatory factor X8 (*RFX8*) genes, respectively, on chromosomes 9 and 10 (Table 2). Genes such as *MSRB3* (42), *THOC1* (43), *ASIC2* (44), and *RFX8* (45) are reported to have either neuropathological or neuronal functions. These genes near significantly associated variants are indicated in Figure 2A. Other loci were located in genes that are not functionally annotated or were located in intergenic regions and away from genes. For instance, two associated regions on CFA10 were located around 8.1 and 8.6 Mb, and the closest genes in these regions were long-term synaptic facilitation protein (*LLPH*) and *LOC111097584*.

**Figure 3:**
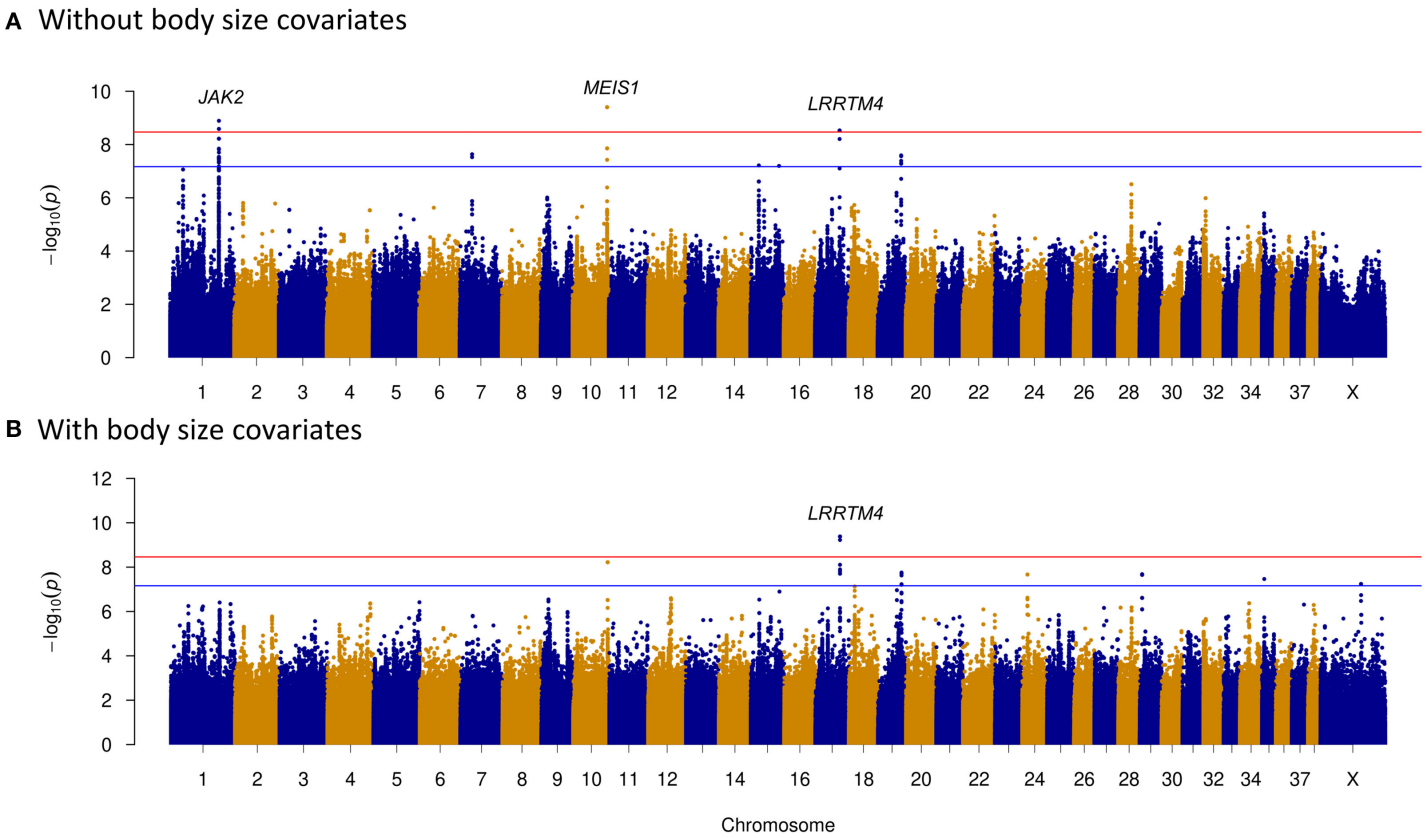
Genome-wide association study (GWAS) of dog predation analysis between hound and herding group dogs. Manhattan plots demonstrate the *p*-value distribution across all chromosomes. The Bonferroni and suggestive GWAS significance thresholds are indicated with the red and blue horizontal lines, respectively. **(A)** Without including body size as a covariate. *JAK2, MEIS1*, and *LRRTM4* were the genes nearest to the significantly associated regions of CFA1, CFA10, and CFA17. **(B)** Including body size as a covariate. Significantly associated region of CFA17 remained after incorporating body size covariates into GWAS.

Prey drive is the innate behavioral pattern of carnivores to pursue and capture prey, and it is a fundamental characteristic of herding dogs. Through selective breeding, humans have been able to reduce prey-driven behavior of herding dogs while maintaining their hunting skills (46). Therefore, we investigated the genetic difference between herding and hunting dogs. Thirty-six hound group dogs and 55 herding group dogs were selected to study the predation differences between these two groups. This may provide further understanding of formation of herding behavior. Three chromosome regions on CFA1, 10, and 17 showed significant signals (Figure 3A). Three genes nearest to these regions were janus kinase 2 (*JAK2*) (about 1 kb), meis homeobox 1 (*MEIS1*) (around 26 kb), and leucine-rich repeat transmembrane neuronal 4 (*LRRTM4*) (~313 kb) (Table 2 and Figure 3A).

**Figure 4:**
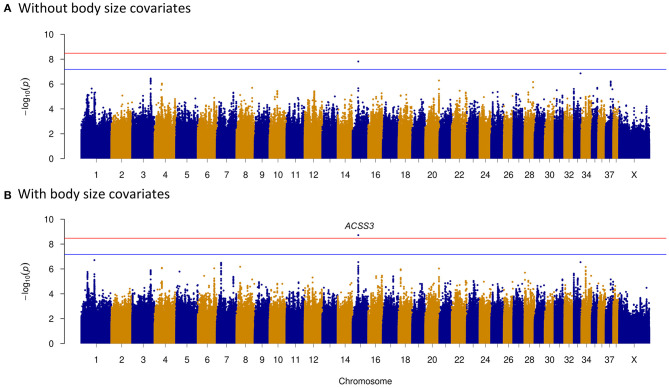
Temperament genome-wide association study (GWAS) reveals an intron variant (CFA15:23,340,008 A>T) of *ACSS3* gene. Manhattan plots showing the association of whole genome variants with temperament levels in dogs. Bonferroni and suggestive thresholds are indicated with red and blue lines. **(A)** Without including body size as a covariate. The ACSS3 intron variant is above the suggestive threshold. **(B)** Including body size as a covariate. The ACSS3 intron variant is above the Bonferroni threshold.

To clarify potential genes that are associated with dog temperament and trainability traits, phenotypes based on breed-averaged measures were grouped as described on the AKC website (https://www.akc.org). The phenotypes were classified into five levels (Figure 1). The GWAS for dog temperament trait was based on 105 dogs of extraversion type and 81 dogs of aloof type. There was only one variant above the suggestive threshold located in the intron region of Acyl-CoA synthetase short chain family member 3 (*ACSS3*) gene on CFA15 (Table 2 and Figure 4A). For trainability GWAS, 98 high and 85 low trainability-level dogs were selected for analysis, and only one variant was detected near *LOC111091672* with a suggestive significant association for trainability (Table 2).

#### Genome-Wide Association Studies With Body Size as a Covariate

**Figure 5:**
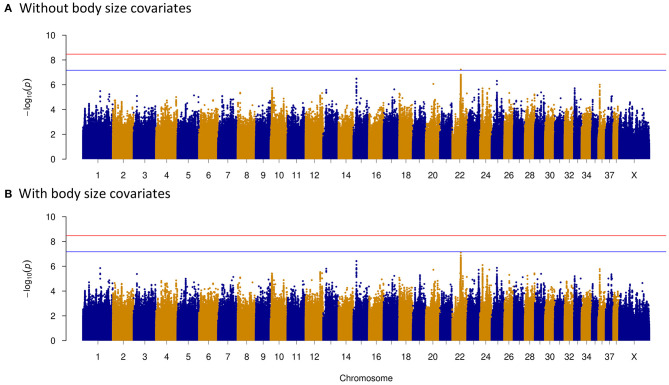
One variant is above the suggestive threshold of dog trainability genome-wide association study (GWAS). Manhattan plots showing the association of whole genome variants with trainability levels in dogs. Bonferroni and suggestive thresholds are indicated with red and blue lines. **(A)** Without including body size as a covariate. One variant on CFA15 slightly passed the suggestive threshold. **(B)** Including body size as a covariate. No variants were detected above the suggestive threshold.

As body size has been reported to be related with dog behaviors, we then performed GWAS adding body size values into covariates. As shown in Figures 2–5, similar results were observed after incorporating SBW and SBH into analysis for herding, predation, temperament, and trainability. In the new herding GWAS, the significantly associated chromosome regions were similar to the results without body size covariates, except for the associated site on CFA9. Although the *p*-values (*p* = 4.03E−09) for the CFA9 variants (CFA9:40067785 and CFA9:40068138) increased, they were still close to the Bonferroni threshold (Figure 2B). Bonferroni significantly associated signals of dog herding behavior around candidate genes like *THOC1, MSRB3, LLPH, RFX8*, and *CHL1* (Figure 2B). For the predation GWAS analysis, only the region of CFA17 remained significantly associated after incorporating body mass covariates into analysis, while variants near *MEIS1* gene on CFA10 were above the suggestive threshold (Figure 3B). In the new temperament GWAS analysis, the same variant of *ACSS3* showed a smaller *p-*value (1.92E−09) above the Bonferroni threshold (Figure 4B). No significant association was found for trainability after adding body size factors. GWAS QQ plots can be referred to Supplementary Figure 1.

#### One Missense Variant of MSRB3 Was in High Linkage Disequilibrium Level With Herding Genome-Wide Association Study Associated Sites

Causative variants are usually not directly detected by GWAS, and phenotypically based causal variants may be in LD with GWAS-related markers. LD of each GWAS significant association site for herding behavior was calculated by Plink 1.90 (31). The results are shown in Supplementary Table 2. Only sites with *r*^2^ > 0.8 were chosen for further analysis. Except variants that already exceeded the significant threshold, six other variants within genes are shown in Table 3. A variant on CFA6 (CFA6:39,977,184 G>A) was located in the intron region of *PIGQ* gene, which has been reported to be the causative gene for human early-onset epilepsy (47). One missense variant (NC_006592.3:g.8037693G>A, XP_013972688.1:p.Gly179Ser) was detected in *MSRB3* gene, and the other four variants were located in exon regions of one lncRNA (*LOC111097584*) near *MSRB3* (38 kb downstream). These five potentially functional variants may promote the development of dog herding behavior through directly or indirectly affecting the functions of *MSRB3* and *LOC111097584*. Ten species were chosen to analyze the *MSRB3* missense variant conservation. Six mammals have amino acid D; and three species including dogs, chickens, and chimpanzees have G in this position (Supplementary Figure 2). This indicated that the missense variant is not conserved.

**Table 3:** Interesting linkage disequilibrium (LD) sites of herding genome-wide association study (GWAS) significant variants with *r*^2^ > 0.8.

**Chromosome**	**Position A**	**Position B**	***r*^**2**^**	**Gene**	**Gene region**	**Gene type**
6	40747205	39977184	0.805667	*PIGQ*	Intron	Protein coding
10	8016660	8037693	0.934985	*MSRB3*	Exon^a^	Protein coding
10	8016660	8079815	0.908056	*LOC111097584*	Exon	LncRNA
10	8016660	8079868	0.912205	*LOC111097584*	Exon	LncRNA
10	8016660	8082492	0.891195	*LOC111097584*	Exon	LncRNA
10	8016660	8083437	0.917264	*LOC111097584*	Exon	LncRNA

*Position A is the position of herding GWAS significant association sites, and position B is the position of LD sites*.

a *One missense mutation within MSRB3: NC_006592.3:g.8037693 G>A, XP_013972688.1:p.Gly179Ser*.

#### Neural Development Processes Were Highlighted in Herding Private Genes With Possible Functions

Private functional variants that were only present in herding dogs could contribute to the herding behavior trait formation. Therefore, we analyzed the private functional genes of herding dogs in an attempt to find candidate genes. To obtain variants that were only present in 55 dogs with herding behavior, high-quality variants of 55 herding and 213 control dogs were separately filtered. After different sites were compared between cases and controls, the variants that only existed in 55 herding dogs were annotated by SnpEff 5.0 software (37). The remaining 7,351 private (611 high impact and 6,740 moderate) variants were chosen for further analysis, and these private variants were in 2,287 protein-coding genes (Supplementary Table 3). Considering that functional variants can impact gene function, these 2,287 genes were used in the GO analysis. Among the top 10 significantly enriched biological processes, 112 genes were enriched in nervous system development process (GO:0007399), and 54 genes were in neuron projection development process (GO:0031175) (Table 4). Moreover, 76 genes were enriched in neuron part (GO:0097458) within cellular component analysis. Details of the gene names and private functional variants are listed in Supplementary Table 3.

**Table 4:** GO analysis for potentially functional private genes of herding dogs.

**GO category**	**GO ID**	**Description**	***p*-value**	**FDR *p*-value**	**Gene number**
Biological process	GO:0051239	Regulation of multicellular organismal process	3.49E−6	1.74E−2	167
	GO:0120036	Plasma membrane bounded cell projection organization	5.65E−6	1.74E−2	82
	GO:0050793	Regulation of developmental process	9.18E−6	1.89E−2	138
	GO:0030030	Cell projection organization	1.32E−5	2.04E−2	82
	GO:0045595	Regulation of cell differentiation	4.66E−5	5.75E−2	95
	GO:0007399	Nervous system development	5.86E−5	6.03E−2	112
	GO:2000026	Regulation of multicellular organismal development	7.00E−5	6.20E−2	109
	GO:0031175	Neuron projection development	9.99E−5	7.70E−2	54
	GO:0048869	Cellular developmental process	1.88E−5	0.11	193
	GO:0030154	Cell differentiation	1.95E−4	0.11	184
Cellular component	GO:0044463	Cell projection part	1.40E−09	5.54E−7	73
	GO:0120038	Plasma membrane bounded cell projection part	1.40E−09	5.54E−7	73
	GO:0044459	Plasma membrane part	1.08E−08	2.85E−6	129
	GO:0042995	Cell projection	2.76E−08	5.11E−6	98
	GO:0005886	Plasma membrane	3.22E−08	5.11E−6	216
	GO:0120025	Plasma membrane bounded cell projection	4.09E−08	5.40E−6	96
	GO:0071944	Cell periphery	4.95E−08	5.61E−6	220
	GO:0098590	Plasma membrane region	2.52E−06	2.50E−4	55
	GO:0097458	Neuron part	3.22E−06	2.84E−4	76
	GO:0005887	Integral component of plasma membrane	5.92E−06	4.70E−4	71

*GO, gene ontology; FDR, false discovery rate*.

#### Nine Candidate Genes Were Highly Expressed in Different Cell Stages of Neural Development Process

As these genes are related to nervous system or human mental disorders, 10 candidate genes, i.e., *THOC1, ASIC2, LLPH, RFX8, MSRB3, CHL1, JAK2, MEIS1, LRRTM4*, and *ACSS3*, were used for further analysis in a single-cell expression database of human neural developmental. Except *ASIC2*, nine candidate genes were detected to be highly expressed in different early development stages of neural cells, which were generated after 12, 26, 54, and 80 days' differentiation (Supplementary Figure 3). It is noted that *RFX8* gene showed unique high expression in neural cells of 12 days.

After these 10 genes were checked in Allen Developing Mouse Brain Atlas, three genes including *ASIC2, CHL1*, and *MEIS1* showed high expressions in mouse brain development stages (E11.5, E13.5, E15.5, E18.5, P4, P14, and P28). This suggests that *ASIC2* is also a neurodevelopmental gene.

## Discussion

Research on the genetic mechanism of dog behaviors can help us understand dog domestication process and guide us on how to get along with dogs, which is important for dog welfare. Moreover, it could also provide clues to research of human behavior and health disorders. Dog genomes have undergone strong artificial selection with increased haplotype homozygosity and LD (2). Therefore, compared with human studies, GWAS with smaller dog samples can even produce good results (2, 15). For example, GWAS with whole-genome sequences across diverse breeds has proved to be a powerful method to study canine morphological traits (5). Here, we used genomic data from 268 modern dogs to perform GWAS for four behaviors and tried to find the genetic clues behind these phenotypes. In this study, phenotypes were based on dog breed standard values or group information from the AKC, which is valid for revealing genomic regions and variants for several specific phenotypes such as dog fear, aggression, boldness, cognition, and athleticism (4, 6, 7, 9, 24). Some dog behaviors have been reported to be highly heritable and higher than those assessed within breeds, and it is hypothesized that specific loci associated with behavioral differences between breeds can be found using across-breed genome-wide approach (7). Previous behavior or cognition GWASs were all performed with SNP chip data (≤173 K), whereas we used nearly 15 M variants of 130 dog breeds in this study, which were obtained by whole genome resequencing. It has higher coverage of non-coding regions of the dog genome, which have important roles in dog behavioral traits such as differentiating dog from wolf (48). In this study, several promising candidate genes with neuronal or psychiatric were detected to be associated with breed differences of herding, predation, temperament, and trainability traits.

Herding is a complex behavioral trait that requires dogs to be fearless and bold when facing large numbers of sheep or cattle. The genome-wide significant loci of fearless were mapped on CFA7:75–79 Mb and CFA20:8–11 Mb (13), and those of boldness were discovered on CFA10:6.8–8.8 Mb (4). In our herding GWAS results, nearby genomic regions of 67.1 Mb on CFA7 and 16.6 Mb on CFA20 were detected to be significantly associated (Table 2). Furthermore, two regions of 8–8.1 and 8.6 Mb on CFA10 were also significantly related. These regions were either near or in the regions that were reported with dog behaviors before. The area of CFA10:8–8.6 Mb has been found to be associated with at least two morphological (ear type and body size) (3, 5, 24) and two behavioral (boldness and fear) traits (4, 7), including genes such as *MSRB3* and *HMGA2*. *MSRB3* has been reported to be associated with human deafness (42, 49), brain morphology, and late-onset Alzheimer's disease (50). It is also involved in stress resistance in *Drosophila* (51). Furthermore, according to GWAS Catalog database (https://www.ebi.ac.uk/gwas/genes/MSRB3), *MSRB3* was detected to be significantly associated with brain area volumes with the largest number of associations among the 31 reported traits. These reports suggest that *MSRB3* gene plays multiple roles in the nervous system. Except *MSRB3* region, we also identified a fragment downstream of *HMGA2*, which is closer to the *LLPH* gene. It has been reported that *LLPH* is involved in regulating neuronal development and synaptic transmission (52). Ear shape and body mass are two common targets of selection in domestic breeding, and selective breeding for specific traits in dogs may result in this region being selected. Also, body size was investigated to be correlated with dog behaviors (11), which were also observed in several genome-wide mapping of dog behaviors (4, 8, 24). One plausible explanation for these associations could be pleiotropy of these regions, which implies that genetic variants could affect both behavior and morphology traits in dogs. Alternatively, morphological and behavioral traits may have been co-selected due to genetic linkage (53).

We also localized another region on CFA10 (41.5 Mb) that was associated with herding behavior, which covered exons 9 and 10 of the *RFX8* gene (Table 2). This region is ~1.99 Mb apart from the top significantly associated site (CFA10:43493767) of dog rivalry behavior (7). It was suggested that *RFX8* could play roles in Schwann cell proliferation, as it was detected to be most prominently expressed in the schwannoma cell line (45). Schwann cells are important for the nervous system, as they direct the regeneration of peripheral axons (54). Meanwhile, *RFX8* has been identified as a candidate gene underlying human neurodevelopmental disorders (55). A significantly associated region on CFA20 covered the uncharacterized *lncRNA-LOC111091431*, the closest to which is a neural-associated gene, *CHL1*. LncRNAs are thought to be commonly but not absolutely involved in transcriptional regulation of nearby genes and often function as cis in enhancer activity (56). Thus, it is assumed that *LOC111091431* may influence the formation of behavior through unknown interactions with *CHL1*, but its exact function remains to be verified. It was reported that *CHL1* could promote neurite outgrowth (57) and regulate cell migration during nerve regeneration (58). It is suspected that *CHL1* is also associated with intelligence (59); this could be an explanation of the higher learning ability of herding dogs. Meanwhile, *CHL1* was detected to be significantly associated with dog fear (7) and human 3p syndrome mental impairment (60). Mice with *CHL1* deficiency demonstrated exploratory behavior changes in novel environments (61) and affected several behavioral parameters such as emotional reactivity (stress) and motor coordination (62). It was also supposed that *CHL1* could participate in nervous system development and signal transduction by regulating synaptic vesicle recycling (63).

In addition to requirements of courage, herding dogs have hunting instincts such as chasing. They are CCD-like behavioral traits that are manifested by dogs using pacing and circling to maintain and control the herd. Some CCD behaviors derive from predatory behavior, like tail chasing and fly snapping (15). The same study reported a strongly associated region of CCDs between 61.83 and 63.87 Mb on CFA7, including *CDH2* gene (15, 16). It is noted in our findings that the region significantly associated with herding was localized between 67.13 and 67.16 Mb on CFA7, ~3.26 Mb from the above-mentioned CCD interval. In addition, a significantly related variant CFA7:67137186 T>G was only 594 bp upstream of *THOC1* gene. However, the abovementioned variant is located within 27 Ts in a row, which suggests that it is unlikely to be regulatory. It is noted that *THOC1* gene is involved in presynaptic development and plays roles in dopamine neuron survival (43). It is also one causative gene for human late-onset hearing loss (64). Herding dogs have been selectively bred to detect and react to slight differences in whistle commands from a long distance of nearly 1 km, and excellent hearing ability is necessary for herding tasks (65). Therefore, genes that are essential for auditory functions such as *MSRB3* and *THOC1* were detected in our herding GWAS analysis.

Significantly associated regions of herding GWAS were also mapped on CFA9 containing *ASIC2* gene. *ASIC2* was reported to play roles in hippocampal neurons (44) and innate fear-like behaviors in mice (66). GO analysis revealed that *ASIC2* was detected in multiple neural cell components (Table 4). *ASIC2* was also detected among private genes of herding dog (Supplementary Table 3). *ASIC2* was detected in high expressions in mouse brain development processes. However, significant signals were absent in the GWAS analysis including body mass factors (Figure 2B). Though gene functions of *LOC611691* and *OR28H03* detected on CFA6 were not related with neural function, one high LD site with the associated variant was located within *PIGQ* gene (Table 3). It has been reported that *PIGQ* is associated with the neurologic disorder of severe early-onset epilepsy (47). Overall, genes *MSRB3, LLPH, RFX8, CHL1, THOC1*, and *ASIC2* are our top candidates based on herding GWAS and likely functional variation in behavioral genes.

Hunting dogs exhibit higher prey-driven behavior in orientation, chasing, grab-bite, and kill-bite (67). They usually show more excitement and aggression when hunting. However, herding dogs have higher abilities of eye-stalk and chase but strongly inhibit the grasping, biting, and killing instincts to prevent them from hurting livestock (19, 21). In a study of the behavioral interactions between dogs and livestock during herding, dog lip-licking and barking occurred less frequently, while stalking, crouching, and chasing were more frequent. Moreover, not a single case of biting was observed (21). Different neurotransmitters have been detected among three dog breeds with distinct predatory behaviors: Border Collies, Siberian Huskies, and Sharplaninatz (68). The GWAS between hound and herding dogs revealed three genes for prey-driven behavior (Figure 3A). The *JAK2* gene is located 1,193 bp downstream of CFA1 association region (Table 2), which has been previously detected to be associated with dog snout ratio and curly tail (3, 4). One study found that dog chasing behavior has been significantly associated with skull shape. Specifically, hound or herding dog breeds tend to have long skulls as their historical roles in pursuit of potential prey animals or livestock, while companion dogs such as toy group canines tend to have short skulls. It implies that skull shape is an indicator of hunting-related behavior (11). Artificial selection based on morphological traits (like short skulls) could have affected dog behavior traits (like tendency to hunt). Meanwhile, *JAK2* is widely expressed and found to be potentially associated with dozens of traits by GWAS Catalog (https://www.ebi.ac.uk/gwas/genes/JAK2). Among these diverse roles, *JAK2* gene is involved in synaptic plasticity and has an essential role in the induction of NMDA-receptor dependent long-term depression (69). Inactivation of *JAK2* can cause memory loss in Alzheimer's disease (70). We found that *MEIS1* gene was detected as the nearest gene to the significantly associated region on CFA10 (Table 2 and Figure 3A), and *MEIS1* was reported to be associated with restless legs syndrome (71). Patients with this neurological disorder have an irresistible urge to move their legs, which can affect sleep quality and even cause mood problems, like depression. Hyperactivity was also observed in heterozygous *MEIS1*-deficient mice, suggesting its role in the specification of neuronal progenitors (72). Therefore, we propose that *MEIS1* may be associated with greater search and chase impulses in hounds when confronted with prey. The nearest gene to the significantly associated region on CFA17 for predation was *LRRTM4*. It has been reported that *LRRTM4* facilitates formation of excitatory synapse development on hippocampal dentate gyrus granule cells (73). More importantly, this gene was close to the strongest associated signal in GWAS analysis of children with aggressive behavior (74). Combined with the gene function and the report in humans, we suggest that *LRRTM4* may play a role in the differences in aggressive behavior between hounds and herding dogs. Moreover, only *LRRTM4* gene was left to be significantly associated with predation after correcting for breed standard body sizes.

Well-behaved dogs are appealing and conducive to establishing good interaction with humans. Temperament and trainability are the foundation of a dog's daily socialization or sports training, which are interesting traits for both dog owners and breeders. Dog fetching behavior has been detected to have suggestive association with CFA22:32270336, which is 2.6 Mb away from our significantly associated signal CFA:34873149 (12). Fetching behavior has been proven to be the most efficient training method for building human–dog relationships, and it is a good indicator of trainability. The significantly associated gene of temperament GWAS has been detected to be *ACSS3* gene (Figure 4). Recently, *ACSS3* has been reported to be significantly associated with human depressive symptoms (75) and antidepressant response (76). Our results suggested that the *ACSS3* gene may contribute to the development of temperament in dogs. Different breeds of dogs have been strongly artificially selected to perform different tasks, accompanied by the production of multiple personalities. Increasing numbers of researches are focusing on the possibilities of dogs as models for studying neurological diseases (29, 77). Although the variant significantly associated with dog trainability is nearest to *LOC111091672*, the nearest protein-coding genes upstream and downstream are *SPRY2* (distance of 1.50 Mb) and *SLITRK1* (distance of 1.45 Mb). *SPRY2* was detected to be highly expressed in the human brain, with the highest expression in the cerebellum [http://biogps.org/#goto=genereport&id=10253; (78)]. Variants in *SLITRK1* gene are associated with human psychiatric disorders such as Tourette's syndrome (79) and OCD (80). MacLean et al. found that trainability had a very high heritability (h^2^ = 0.73) (7), indicating that the percentage of variance explained in the GWASs should be high. However, our top vs. bottom GWAS designed based on the AKC breed standard descriptions was underpowered. The AKC written descriptions of dog breed temperament and trainability are not accurate enough for detecting variants controlling the behavioral differences among breeds. This may be one reason for the less signals obtained in GWAS of temperament and trainability.

The selection of genomic regulatory regions could contribute large effects on the formation of canine breed standards (81). Notably, epigenetic variations also play important roles in the behavioral formation (82–84). This might due to the fact that gene coding regions are more conserved than non-coding regions, and protein-coding regions typically evolve at a slower rate. Behavioral selection for dog domestication might be caused by the regulation of gene expressions in the hypothalamus (85). Several variants within lncRNAs or potential gene regulatory regions were detected in our studies, which implies that they could play crucial roles in herding behavior formation through regulating gene expressions of the candidate neural genes.

Enrichment analysis was performed with candidate genes obtained from private variant analysis. Several processes or cellular components related to neurology function were obtained (Table 4). This indicates that changes in the regulation of neuron and nervous system development could contribute to herding behavior formation. These seven candidate genes could be involved in the early neural system development (Supplementary Figure 3), which raises their possibilities of being regarded as candidate genes underlying dog behaviors. To increase the credibility of mapping, only variants above the Bonferroni genome-wide significance threshold were considered as candidates for herding and predation GWAS. Overall, seven promising candidate genes were identified for dog herding (*THOC1, MSRB3, LLPH, RFX8*, and *CHL1*), predation (*LRRTM4*), and temperament (*ACSS3*) between dog breeds after correcting with body mass in this study. Though associations of *ASIC2, JAK2*, and *MEIS1* gene regions were not above significant threshold after controlling body size, they could still have potential roles on dog behaviors through effects on dog brain architectures, which are related with body mass.

There are several limitations in this study. Specifically, herding group dogs are from different breeds that share herding behavior, but we were not able to determine if all herding dog breeds share a common ancestor. Phenotypic classification based on breed standard described by the AKC is not robust enough to detect all the genetic variants between dog breeds, especially for trainability. Further studies using breed-average C-BARQ values could improve the accuracy. Even though GWASs using small numbers of dog individuals of very many breeds have proved to be powerful methods to identifying variants influencing morphology (5), it is still prudent to simply apply GWASs to behavioral traits.

To fine-map the casual variants or genes for these behavioral traits accurately, professional behavioral scientists are required to perform accurate phenotypic dissections for those traits, which will be performed in Dog10K project (86). With more accurate phenotypic definitions of dog behavioral traits and more dog whole-genome sequences released by Dog10k project, the understanding of genetic mechanisms underlying these behavioral traits will be significantly enhanced. In the following studies, accurate behavioral measurement methods such as Herding Trait Characterization (HTC) could be applied to evaluate a large number of dogs from diverse breeds (87). Similar to this study, GWASs using breed-average scores of HTC questionnaire as phenotypes can be applied to identify genetic differences among dog breeds. To improve genetic mapping accuracy and reveal additional genes for these four dog behaviors, GWASs can be performed using genotype and phenotype data from the same canine individuals.

Cross-breed mapping approaches can effectively identify loci that may affect genetic differences between breeds that cannot be studied by segregation within breeds. The classic example is that the specific negative correlation between longevity and size is a strictly between-breed phenomenon and is difficult to conduct genetic analysis by within-breed studies (24). The herding behavior is also a clear between-breed behavior. Therefore, the method of classifying behaviors according to the historical roles of dogs and analyzing herding behaviors among dog breeds is reasonable. This was also reflected in the genetic mapping of herding, pointing, boldness, and athleticism in dogs, and convincing genes appropriate to behaviors were obtained (4, 9, 24). Zapata et al. (77) performed a genome-wide scan of several dog behaviors of diverse breeds and also identified genes that overlap with human neurodevelopmental and psychopathological genes, implying that dogs and humans share some degree of common molecular mechanisms during neurological development. Hence, this study may provide genetic clues to further elucidate the formation of behavioral traits in dogs and provide potential models for studying complex neuropsychiatric disorders in humans.

## Data Availability Statement

The original contributions presented in the study are included in the article/Supplementary Material, further inquiries can be directed to the corresponding author/s.

## Author Contributions

SS: data analysis, writing-original draft, review, and editing. FX: conceptualization, data analysis, writing-original draft, review, and editing. BB: supervision, writing-review, and editing. All authors contributed to the article and approved the submitted version.

## Conflict of Interest

The authors declare that the research was conducted in the absence of any commercial or financial relationships that could be construed as a potential conflict of interest.
