# Supplementary figures and images for: Genome-Wide Association Studies Reveal Neurological Genes for Dog Herding, Predation, Temperament, and Trainability Traits
Source: Front Vet Sci. 2021 Jul 21;8:693290. doi: 10.3389/fvets.2021.693290 (PMC8335642; doi:10.3389/fvets.2021.693290)

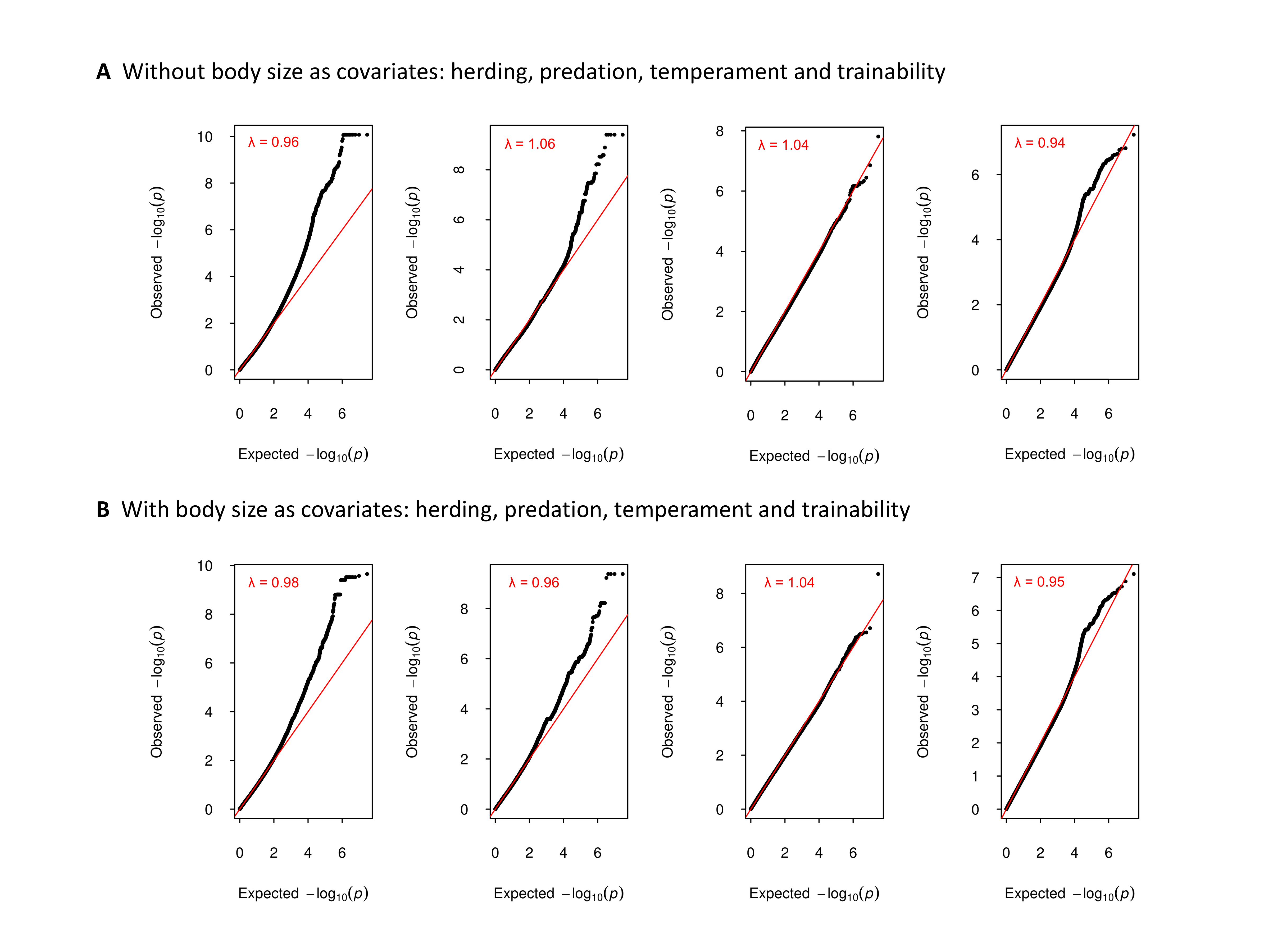

Supplement: Supplementary file 1 [file Image_1.JPEG]

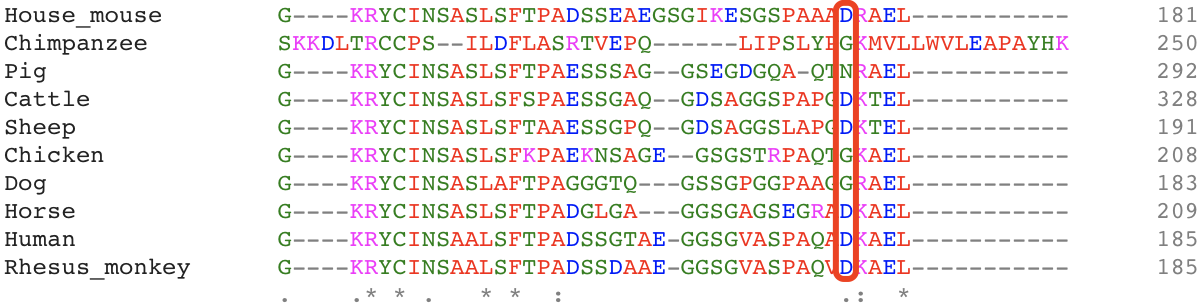

Supplement: Supplementary file 2 [file Image_2.TIFF]
