# Supplementary material for: Genome-Wide Association Studies Reveal Neurological Genes for Dog Herding, Predation, Temperament, and Trainability Traits
Source: Front Vet Sci. 2021 Jul 21;8:693290. doi: 10.3389/fvets.2021.693290 (PMC8335642; doi:10.3389/fvets.2021.693290)
Supplement: Supplementary file 7 [file Table_4.docx]

**Supplementary information**

**Legends for the supplementary figures and tables**

**Supplementary Figure 1**: QQ plots of dog herding, predation, temperament and trainability GWASs without **(A)**/with **(B)** including body size as covariates.

**Supplementary Figure 2**: Evolutionary conservation analysis results of *MSRB3* missense mutation in 10 species using Clustal W (<https://www.ebi.ac.uk/Tools/msa/clustalo/>). The amino acid sequence accession numbers of the 10 species are as follows: House mouse XP_006513829.1, Chimpanzee XP_016778576.1, Pig XP_020947614.1, Cattle XP_024848255.1, Sheep XP_027823281.1, Chicken XP_015137097.1, Dog XP_013972688.1, Horse XP_023499721.1, Human XP_024304686.1 and Rhesus monkey NP_001244780.1.

**Supplementary Figure 3**: Single-cell gene expression of 10 candidate genes through the neural development processes (oocyte, zygote, 2-cell, 4-cell, 8-cell,16-cell, blastocyst, human embryonic stem cells (hESC), H1_24_wells, H1_96_wells, neural_D12 neural_D26, neural_D54, neural_D80). Dn: cell line fused with endogenous SOX2 (a marker of progenitors) Dp: cell line fused with DCX (a marker of immature neurons).

**Supplementary Table 1.** Phenotypic information of 268 dogs. Case/control/missing are indicated by 2/1/NA.

**Supplementary Table 2.** Linkage disequilibrium sites of herding GWAS significant association sites (r^2^ > 0.8).

**Supplementary Table 3**. 2287 potentially functional private genes and priority candidate variants of herding dogs.
